# Association Between Physical Activity Levels and Sleep Quality Among Saudi Board Trainees: A Cross-Sectional Study

**DOI:** 10.7759/cureus.104447

**Published:** 2026-02-28

**Authors:** Amal Radi A Aljuhani, Abdulhameed A Alharbi

**Affiliations:** 1 Preventive Medicine, Al-Suraif Primary Health Care Center, Al-Madinah Health Cluster, Ministry of Health, Yanbu, SAU; 2 Preventive Medicine, Preventive Medicine Postgraduate Program, Al-Madinah Health Cluster, Ministry of Health, Al-Madinah, SAU

**Keywords:** internship, residency, saudi arabia, sedentary behavior, sleep quality

## Abstract

Background

Medical residency training is associated with high workload, psychological stress, and irregular sleep schedules, all of which may negatively affect sleep quality and mental well-being. Although physical activity is known to improve sleep and psychological health, limited evidence exists regarding its independent association with sleep quality among Saudi board trainees.

Aim

This study aimed to estimate the prevalence of poor sleep quality and identify its predictors among Saudi board trainees.

Methods

This cross-sectional survey study used an online, self-administered questionnaire that covered participants’ demographics and personal characteristics. Sleep quality was assessed using the Pittsburgh Sleep Quality Index (PSQI), physical activity was evaluated using the short form of the International Physical Activity Questionnaire (IPAQ-SF), and anxiety and depression were screened using the four-item Patient Health Questionnaire (PHQ-4). Logistic regression models were applied to identify factors associated with poor sleep quality.

Results

The prevalence of poor sleep quality was 51.5% (101/196). Univariable logistic regression analysis revealed that several factors were significantly associated with increased odds of poor sleep quality, including being a junior resident (OR: 1.188, 95% CI: 1.032-1.366, p=0.017), use of sedative medications (OR: 1.640, 95% CI: 1.327-2.027, p<0.001), anxiety (OR: 1.482, 95% CI: 1.148-1.913, p=0.003), depression (OR: 1.495, 95% CI: 1.104-2.024, p=0.010), and mixed anxiety and depression (OR: 1.757, 95% CI: 1.482-2.083, p<0.001). However, in the multivariable logistic regression model, only the use of sedative medications and the presence of anxiety and/or depression remained independently associated with poor sleep quality. Residency level and physical activity did not retain statistical significance.

Conclusions

Anxiety and depression emerged as the most significant factors associated with poor sleep quality among Saudi residency trainees. Although physical activity initially appeared protective, it was not independently associated with sleep quality after adjusting for other relevant variables. Further research is needed to better understand the underlying mechanisms and consequences of anxiety and depression to guide strategies for improving sleep and well-being among Saudi residents.

## Introduction

The period of medical residency is a highly demanding phase owing to long working hours, high levels of stress, and sleep deprivation, all of which represent substantial challenges that may negatively impact residents' well-being and performance [1]. The Saudi Commission for Health Specialties (SCFHS) is responsible for supervising a wide array of residency training programs across different medical fields. It is committed to enhancing the quality of training and promoting the well-being of residents to develop a strong healthcare workforce capable of effectively meeting the evolving healthcare needs of the Saudi population [2,3].

Medical residents exhibit high vulnerability to sleep disturbances. Among junior clinicians, sleep deprivation is significantly correlated with burnout, depressive symptoms, and diminished quality of life. It is also associated with an increased incidence of major medical errors and a higher risk of malpractice litigation [4]. Research conducted in Saudi Arabia highlights a significant prevalence of sleep disturbances among medical students. One study reported that nearly one-third of students exhibited abnormal sleep patterns, necessitating urgent intervention [3]. Further investigations have shown that poor sleep quality affects a majority of healthcare students and is positively correlated with elevated levels of stress, anxiety, and depression [5].

Several approaches can promote healthy sleep habits, including education on sleep hygiene, optimization of the sleep environment, and the use of pre-bedtime relaxation techniques. Additionally, improvements in working conditions, such as limiting prolonged shift durations and ensuring adequate rest periods between shifts, can substantially reduce sleep deprivation and occupational fatigue. Regular physical activity is a particularly effective strategy for enhancing sleep quality, as it promotes relaxation, reduces stress and anxiety, supports circadian rhythm regulation, and increases the proportion of restorative deep sleep [6-9].

A substantial body of evidence demonstrates a strong association between physical activity, sleep quality, and mental health, highlighting the beneficial role of physical activity in enhancing sleep and psychological well-being [3,5,10-12]. Despite this, research examining these relationships in specific high-risk populations remains limited. Medical trainees, particularly those enrolled in Saudi board residency programs, experience intensive workloads and unique occupational stressors that may adversely affect their sleep and overall health; however, their sleep quality and physical activity patterns have not been adequately explored. Investigating these factors in this population is essential, as insufficient sleep and low levels of physical activity may compromise both quality of life and clinical performance. Accordingly, this study aims to determine the prevalence of poor sleep quality and levels of physical activity among SCFHS Board trainees and to examine the association between physical activity and sleep quality.

This study is informed by the biopsychosocial model, which posits that physical activity influences sleep quality and mental health through interconnected biological, psychological, and social pathways. Biologically, regular physical activity supports circadian rhythm regulation and hormonal balance, thereby facilitating restorative sleep. Psychologically, physical activity has been shown to alleviate stress and anxiety, which are prevalent among medical trainees due to the demands of residency training. Socially, engagement in physical activity may enhance overall well-being and resilience to occupational stressors [13-15]. Together, this framework provides a comprehensive basis for examining the potential mitigating effects of physical activity on sleep disturbances and mental health among Saudi board trainees.

The primary objective of this study was to estimate the prevalence of poor sleep quality among Saudi board trainees enrolled in programs supervised by the SCFHS. Secondary objectives were to examine the association between physical activity levels and sleep quality and to identify independent factors associated with poor sleep quality, including anxiety, depression, sedative medication use, and training-related variables. The findings may provide an evidence-based foundation to inform targeted interventions aimed at improving trainee well-being and enhancing professional functioning within the Saudi healthcare context.

## Materials and methods

Study design, setting, and duration

This cross-sectional study was conducted in Saudi Arabia between September 1 and October 31, 2024.

Eligibility criteria

The inclusion criteria for the study were adult trainees registered in the SCFHS Board programs who were actively undergoing training during the current training year. Exclusion criteria included pregnant female trainees and those with comorbidities affecting their daily activities.

Sample size calculation and sampling technique

This study was conducted across all sectors of the SCFHS. The target population consisted of approximately 20000 trainees enrolled in SCFHS Board programs. We used a non-probability convenience sampling technique. The sample size was calculated using the single population proportion formula, assuming a prevalence of poor sleep quality of 86.3%, a 95% confidence level, and a 5% margin of error. The minimum required sample size was 181 participants [16]. To account for an anticipated 15% non-response rate due to the online survey design, the final target sample size was increased to 211 participants.

Data collection and study tool

Data collection was performed via a structured online survey hosted on the Google Forms platform. It was administered in English, the official language of medical training in residency programs supervised by the SCFHS. The survey was disseminated to eligible participants through targeted WhatsApp professional groups dedicated to medical residents. The survey’s introductory module comprised a comprehensive informed consent form outlining the study objectives, procedural protocols, potential risks and benefits, and guarantees of confidentiality and voluntary participation. To ensure ethical compliance, explicit digital consent was required before proceeding. Daily monitoring of the platform’s real-time analytics was performed to track response metrics and assess completion rates. To mitigate non-response bias, a follow-up reminder was issued one week post-distribution, with subsequent prompts as required. Prior to formal data export and analysis, a rigorous data-cleaning protocol was implemented to exclude incomplete submissions, duplicate entries, and statistically invalid responses.

The first section of the instrument (Appendix) assessed sociodemographic and clinical profiles, encompassing age, gender, marital status, pregnancy, and nationality, alongside professional data such as training sector, medical specialty, and residency level. Health-related behaviors were quantified using several validated tools. Tobacco use was recorded via the Tobacco Questions for Surveys (TQS) [17] to align with international monitoring standards, while caffeine consumption was evaluated using the Diet History Questionnaire (DHQ) [18], a food frequency instrument designed for adults. Additionally, participants’ medical, surgical, and psychiatric histories were documented, and the use of medications, including melatonin, analgesics, and antihistamines, was assessed. Psychological distress was screened using the Patient Health Questionnaire-4 (PHQ-4) [19]. This valid and reliable scale utilizes a four-point Likert-style range (0-3) to measure symptoms of anxiety and depression. Total scores are aggregated and stratified into four severity levels: normal (0-2), mild (3-5), moderate (6-8), and severe (9-12). Specifically, a sub-score of ≥3 on the first two items indicates potential anxiety, while a similar score on the final two items suggests depression. Research has shown that the PHQ-4 has adequate internal consistency (α=0.65-0.84) and strong test-retest reliability [20].

The second section utilized the Pittsburgh Sleep Quality Index (PSQI) to evaluate subjective sleep quality over the preceding month. This standardized instrument comprises 19 items categorized into seven functional components: subjective sleep quality, sleep latency, habitual sleep efficiency, sleep duration, sleep disturbances, use of hypnotic medication, and daytime dysfunction. Each component is assigned a score from 0 to 3, which is then summed to produce a global score ranging from 0 to 21. For clinical interpretation, global scores of 0-4 signify good sleep quality, 5-10 indicate poor sleep quality, and scores exceeding 10 reflect severe sleep impairment. The PSQI is recognized for its high internal reliability, characterized by a Cronbach’s alpha of 0.83 in diverse populations [21].

Physical activity levels were assessed in the third section using the International Physical Activity Questionnaire-Short Form (IPAQ-SF). This seven-item standardized tool measures the frequency and duration of walking, as well as moderate- and vigorous-intensity activities, performed over the last seven days. Scoring involves the calculation of metabolic equivalent task (MET) minutes per week by multiplying the MET value associated with each activity type by its daily duration and weekly frequency. Based on these cumulative MET-minutes, participants were categorized into low, moderate, or high activity levels, providing an objective measure of adherence to physical activity guidelines. This tool has demonstrated acceptable reliability and validity across multiple countries and is publicly available through the official IPAQ website [22-24].

Data analysis

Statistical analysis was performed using the R statistical language (version 4.4.3; R Foundation for Statistical Computing, Vienna, Austria) [25]. All question responses were treated as categorical variables and summarized as counts and percentages. The association between sleep quality (categorized as good and poor sleep categories based on the total PSQI score) and participants’ sociodemographic and health-related characteristics was assessed using Pearson’s chi-square test for independence of observations or Fisher’s exact test when more than 20% of the expected cell counts were less than five. Univariable and multivariable logistic regression models were used to identify factors significantly associated with sleep quality. A p-value <0.05 was considered statistically significant.

Ethical considerations

This study was approved by the Institutional Review Board (IRB), Health Affairs, Al-Madinah (IRB log No. 24-067; National Registration Number: H-03-M-84). Participants were provided with a clear explanation of the research purpose and were free to accept or decline participation. Informed consent was obtained prior to completion of the study questionnaire. Strict confidentiality was maintained throughout all stages of the research process.

## Results

Sociodemographic and health-related characteristics of the participants

The total number of respondents was 211. We excluded nine female respondents who reported being pregnant and six respondents with medical conditions interfering with their daily activities. The number of participants included in the analysis was 196.

In the present study, participants’ age ranged between 25 and 44 years (mean±SD: 29.2±3.4 years), with 91.3% (179/196) between 25 and 34 years. The percentage of male participants was slightly higher than that of females (53.1% (104/196) vs. 46.9% (92/196), respectively). Most participants were single (119, 60.7%). Nearly all participants (193, 98.5%) were Saudis. More than one-third (72, 36.7%) were from the fourth training sector, while approximately one-quarter (52, 26.5%) were from the second sector. Nearly three-fourths (146/196) specialized in medical fields, while the remaining participants specialized in surgical fields. More than half of the participants (113, 57.7%) were at the senior residency level (Table 1).

**Table 1:** Participants’ sociodemographic characteristics

Characteristics	N=196	%
Age group (years)		
25-34	179	91.3
35-44	17	8.7
Gender		
Female	92	46.9
Male	104	53.1
Marital status		
Married	71	36.2
Single	119	60.7
Separated/divorced/widow/widower	6	3.1
Nationality		
Saudi	193	98.5
Non-Saudi	3	1.5
Training sector		
First (Central region)	34	17.4
Second (Western region)	52	26.5
Third (Eastern region)	17	8.7
Fourth (Northern, Al-Madinah, and Al-Qassim regions)	72	36.7
Fifth (Southern region)	21	10.7
Specialization		
Medical fields	146	74.5
Surgical fields	50	25.5
Residency level		
Junior	83	42.3
Senior	113	57.7

Regarding the level of physical activity, 40.3% (79/196) reported a low level, while 46.4% (91/196) and 13.3% (26/196) reported moderate and high levels of activity, respectively. Half the participants (99, 50.5%) were within the normal range for body weight, while 28.6% (56/196) were classified as overweight and 16.8% (33/196) as obese. The participants' health-related characteristics showed that 141 (71.9%) never smoked, five (2.6%) were ex-smokers, and 50 (25.5%) were current smokers. Moreover, 132 (67.3%) of them consumed caffeine more than once per day. The prevalences of anxiety, depression, and mixed anxiety and depression were 6.7% (13/196), 4.6% (9/196), and 16.8% (33/196), respectively. Medical history was negative in 158 (80.6%), and most of the participants (174, 88.8%) were not using any sedative drugs (Table 2).

**Table 2:** Participants' health-related characteristics NSAIDs: non-steroidal anti-inflammatory drugs; BMI: body mass index

Characteristics	N=196	%
Physical activity		
Low	79	40.3
Moderate	91	46.4
High	26	13.3
BMI		
Underweight	8	4.1
Normal	99	50.5
Overweight	56	28.6
Obese	33	16.8
Smoking status		
Non-smoker	141	71.9
Ex-smoker	5	2.6
Current smoker	50	25.5
Consumption of caffeine-containing beverages		
One time or less per day	64	32.7
>One time per day	132	67.3
Anxiety and depression		
Anxiety	13	6.7
Depression	9	4.6
Mixed anxiety and depression	33	16.8
None	141	71.9
Medical history		
No	158	80.6
Yes, but it does not affect daily activities	38	19.4
Sedatives use		
None	174	88.8
Antihistamines	4	2
Melatonin	8	4.1
Sedative NSAIDs	8	4.1
Melatonin and NSAIDs	2	1

Sleep quality and its associated factors

Based on the total PSQI score, the prevalence of poor sleep among the participants was 51.5% (101/196). We found that a significantly higher percentage of junior residents had poor sleep quality compared to senior residents (61.4% (51/83) vs. 44.2% (50/113)), with junior residents having 1.188 times higher odds of experiencing poor sleep quality (95% CI: 1.032-1.366; p=0.017; Table 3).

**Table 3:** Association between sleep quality and the participants’ sociodemographic characteristics ^*^Significant OR: odds ratio; CI: confidence interval

Characteristics	Good sleep quality, N=95 (48.5%)	Poor sleep quality, N=101 (51.5%)	Unadjusted OR (95% CI)	P-value
Age (years), n (%)				
25-34	88 (49.2)	91 (50.8)	-	
35-44	7 (41.2)	10 (58.8)	1.083 (0.844, 1.390)	0.531
Gender, n (%)				
Female	40 (43.5)	52 (56.5)	-	
Male	55 (52.9)	49 (47.1)	0.910 (0.791, 1.047)	0.19
Marital status, n (%)				
Married	35 (49.3)	36 (50.7)	-	
Single	59 (49.6)	60 (50.4)	0.997 (0.861, 1.155)	0.97
Separated/divorced/widow/widower	1 (16.7)	5 (83.3)	1.386 (0.913, 2.103)	0.127
Nationality, n (%)				
Saudi	93 (48.2)	100 (51.8)	-	
Non-Saudi	2 (66.7)	1 (33.3)	0.831 (0.469, 1.473)	0.528
Training sector, n (%)				
First (Central region)	17 (50.0)	17 (50.0)	-	
Second (Western region)	28 (53.8)	24 (46.2)	0.962 (0.775, 1.195)	0.729
Third (Eastern region)	9 (52.9)	8 (47.1)	0.971 (0.725, 1.301)	0.844
Fourth (Northern, Al-Madinah, and Al-Qassim regions)	29 (40.3)	43 (59.7)	1.102 (0.898, 1.352)	0.353
Fifth (Southern region)	12 (57.1)	9 (42.9)	0.931 (0.709, 1.223)	0.609
Specialization, n (%)				
Medical fields	74 (50.7)	72 (49.3)	-	
Surgical fields	21 (42.0)	29 (58.0)	1.091 (0.929, 1.281)	0.291
Residency level, n (%)				
Junior	32 (38.6)	51 (61.4)	1.188 (1.032, 1.366)	0.017^*^
Senior	63 (55.8)	50 (44.2)	-	

The use of sedative medications was significantly more frequent among participants experiencing poor sleep (95.5% (21/22) vs. 46% (80/174)), with users having 1.640 times higher odds of poor sleep quality (95% CI: 1.327-2.027; p<0.001). The presence of mixed anxiety and depression was significantly associated with poor sleep quality. Participants with anxiety demonstrated significantly higher odds of experiencing poor sleep compared to those without any psychological disorder (OR=1.482; 95% CI: 1.148-1.913; p=0.003). Similarly, participants with depression showed higher odds of poor sleep (OR=1.495; 95% CI: 1.104-2.024; p=0.010). Furthermore, comorbid anxiety and depression were associated with even greater odds of poor sleep (OR=1.757; 95% CI: 1.482-2.083; p<0.001). Regarding physical activity, moderate activity was associated with lower odds of poor sleep (OR=0.858; 95% CI: 0.738-0.996; p=0.046) compared to low physical activity levels (Table 4).

**Table 4:** Association between sleep quality and the participants’ health-related characteristics ^*^Significant OR: odds ratio; CI: confidence interval; BMI: body mass index; WHO: World Health Organization

Characteristics	Good sleep quality, N=95 (48.5%)	Poor sleep quality, N=101 (51.5%)	Unadjusted OR (95% CI)	P-value	
Physical activity level, n (%)					
Low	33 (41.8)	46 (58.2)	-		
Moderate	52 (57.1)	39 (42.9)	0.858 (0.738, 0.996)	0.046^*^	
High	10 (38.5)	16 (61.5)	1.034 (0.829, 1.288)	0.769	
BMI WHO classification, n (%)					
Underweight (<18.5)	4 (50.0)	4 (50.0)	-		
Normal (18.5-24.9)	54 (54.5)	45 (45.5)	0.956 (0.667, 1.369)	0.805	
Overweight (25-30)	26 (46.4)	30 (53.6)	1.036 (0.716, 1.500)	0.85	
Obese (>30)	11 (33.3)	22 (66.7)	1.181 (0.804, 1.737)	0.398	
Smoking status, n (%)					
Non-smoker	68 (48.2)	73 (51.8)	-		
Ex-smoker	2 (40.0)	3 (60.0)	1.086 (0.693, 1.701)	0.72	
Current smoker	25 (50.0)	25 (50.0)	0.982 (0.835, 1.156)	0.831	
Consumption of caffeine-containing beverages, n (%)					
One time or less per day	26 (40.6)	38 (59.4)	-		
>One time per day	69 (52.3)	63 (47.7)	0.890 (0.767, 1.033)	0.127	
History of anxiety and depression, n (%)					
None	88 (62.4)	53 (37.6)	-		
Anxiety	3 (23.1)	10 (76.9)	1.482 (1.148, 1.913)	0.003^*^	
Depression	2 (22.2)	7 (77.8)	1.495 (1.104, 2.024)	0.010^*^	
Mixed anxiety and depression	2 (6.1)	31 (93.9)	1.757 (1.482, 2.083)	<0.001^*^	
Current treatment for depression or anxiety, n (%)					
No	90 (48.4)	96 (51.6)	-	-	
Yes	5 (50.0)	5 (50.0)	0.984 (0.715, 1.355)	0.921	
Medical history, n (%)					
No	77 (48.7)	81 (51.3)	-		
Yes, but it does not affect daily activities	18 (47.4)	20 (52.6)	1.014 (0.849, 1.211)	0.881	
Use of sedative medications, n (%)					
No	94 (54.0)	80 (46.0)	-		
Yes	1 (4.5)	21 (95.5)	1.640 (1.327, 2.027)	<0.001^*^	

A multivariable logistic regression model was developed, incorporating variables that were significant in the univariable analysis. Sedative medication use (p=0.010), depression alone (p=0.048), and comorbid anxiety and depression (p=0.001) remained independently associated with higher odds of poor sleep quality. Conversely, residency level and physical activity were not significant predictors in the multivariable model (Table 5).

**Table 5:** Multivariable logistic regression model to identify significant independent factors contributing to poor sleep ^*^Significant (Nagelkerke’s R2=0.362) OR: odds ratio; CI: confidence interval

Characteristics	Adjusted OR (95% CI)	Wald test	P-value
Residency level			
Senior	Reference		
Junior	1.486 (0.745, 2.957)	1.13	0.258
Use of sedative medications			
No	Reference		
Yes	15.906 (2.866, 298.808)	2.58	0.010^*^
History of anxiety and depression			
None	Reference		
Anxiety	3.453 (0.889, 16.998)	1.7	0.089
Depression	5.300 (1.153, 37.528)	1.98	0.048^*^
Mixed anxiety and depression	19.290 (5.339, 124.056)	3.88	<0.001^*^
Physical activity level			
Low	Reference		
Moderate	0.709 (0.343, 1.463)	-0.93	0.35
High	1.250 (0.443, 3.567)	0.42	0.673

## Discussion

Numerous studies have shown that medical residents are vulnerable to sleep-related problems, which can contribute to reduced cognitive function, decreased alertness, and an increased risk of medical errors [16,26-28]. This study aimed to investigate the relationship between physical activity and poor sleep quality among board trainees enrolled in SCFHS programs. The study provides valuable insights into sleep and physical activity research within the context of medical training in Saudi Arabia.

The results of the present study indicated that the prevalence of poor sleep quality among board trainees was 51.5% (101/196). This result agrees with previous studies from centers in India, Mexico, and Brazil, which reported rates of poor sleep quality among residents of 39.3% [29], 32.1% [30], and 59.3% [31], respectively. On the other hand, previous studies have reported higher prevalence rates of poor sleep quality among medical residents. AlSaif et al. [16] found that 86.3% of 1205 medical residents across Saudi Arabia experienced poor sleep quality, attributing this to work-related stress, irregular shift work, and unhealthy lifestyle habits. In Saudi Arabia, cultural and social obligations, such as extended family duties and late-night gatherings, may further disrupt sleep routines [16]. Religious practices, such as fasting during Ramadan, have also been shown to alter sleep patterns and daytime functioning in healthy individuals [32]. Recognizing these cultural factors is essential for developing culturally sensitive physical activity and wellness guidelines for residents. Similarly, a study among 150 Egyptian medical residents reported a prevalence of poor sleep of 96.7% [33]. Differences in prevalence rates across studies may be attributed to variations in work schedules, work environments, and cultural backgrounds.

Physical activity represents a potentially beneficial intervention to improve sleep quality and general well-being among medical residents. In the current study, physical activity levels were low in 79 (40.3%) trainees, while 91 (46.4%) and 26 (13.3%) had moderate and high levels of activity, respectively. A national study assessing 1616 Saudi adults revealed that many did not meet recommended physical activity levels, in which 40.6% were physically inactive, 34.3% were minimally active, and only 25.1% were considered active [34]. Another study on 194 primary healthcare physicians in Makkah found that 49.4% were inactive and 50.6% were active [11]. These findings highlight the health risks associated with insufficient physical activity among the Saudi population. Our findings are partially consistent with a study conducted in the Al-Jouf region among physicians working in primary healthcare centers, where 44.3% reported moderate and 20.8% reported vigorous physical exercise [35]. Likewise, a cross-sectional study at King Abdulaziz Hospital in Jeddah reported higher rates of vigorous activity compared to our sample, with 36.4% and 41.6% of physicians engaged in vigorous and moderate activity, respectively [36]. The low rates of moderate- and high-intensity exercise observed in the current study and other previous studies may be related to sedentary lifestyles or perceived barriers. The most frequently reported barriers to regular exercise among physicians included lack of time and motivation, as well as limited exercise facilities at home and harsh weather conditions [37,38].

In the univariable analysis, moderate physical activity was associated with lower odds of poor sleep (OR: 0.858, 95% CI: 0.738- 0.996) compared with low physical activity. However, no significant difference was observed between high and low physical activity levels in relation to the odds of poor sleep quality. Meanwhile, physical activity was not significantly associated with poor sleep quality in the multivariable logistic regression model after adjustment for residency level, sedative use, and the presence of anxiety and depression.

Moderate physical exercise potentially improves several components of sleep, including sleep duration, efficiency, and latency. A meta-analysis of 66 studies found that regular moderate-intensity exercise was associated with improvements in both subjective and objective measures of sleep [9]. Likewise, a systematic review of 14 studies showed that moderate-intensity physical activity, rather than vigorous activity, significantly enhanced sleep duration and efficiency while reducing sleep latency in younger and older adults [39].

Previous research has reported mixed findings regarding the effects of vigorous physical activity on sleep quality. A meta-analysis of 38 studies indicated that while some studies demonstrated beneficial effects of vigorous exercise, others suggested potential disruption of sleep patterns [40]. Another study reported that intense physical activity could have mixed effects on sleep quality, with potential adverse effects on sleep architecture if undertaken close to bedtime [41]. A recent systematic review of 23 studies corroborated differences in the effects between moderate- and high-intensity physical exercise [10].

The significant association observed between anxiety, depression, and poor sleep quality may be attributed to their impact on multiple components of sleep. Meanwhile, poor sleep quality can contribute to the severity of anxiety and depression. Previous research has demonstrated a bidirectional relationship between poor sleep, anxiety, and depression [42]. Moreover, a bidirectional genetic relationship appears to exist between physical activity and anxiety and depression [43,44], underscoring the potential role of physical activity in alleviating anxiety, depression, and poor sleep quality among medical residents.

The results of the present study provide important insights into poor sleep quality among medical residents enrolled in SCFHS programs and its major contributing factors. A significant association was observed between moderate-intensity exercise and poor sleep quality in bivariate analysis and univariable regression. However, we were unable to confirm this association in the multivariable model that included several potential risk factors for poor sleep. This lack of statistical significance in the multivariable analysis may be partly attributed to the relatively small sample size of the present study. Another possible explanation for the lack of a significant association is the presence of confounding or effect-modifying factors that were not explored in the current study, such as work schedule and sleep apnea syndrome. Future studies should address these limitations by including an adequate sample size to ensure sufficient statistical power for multivariable regression and by exploring potential confounders and moderators. Future longitudinal or interventional studies, ideally randomized controlled trials, are warranted to clarify the effects of different levels of physical activity on sleep quality.

The present study demonstrated several areas of strength. The study population comprised medical residents from various specialties across Saudi Arabia. A comprehensive investigation was conducted into the potential risk factors associated with poor sleep quality. The measurement of sleep quality and level of physical activity were conducted using validated tools. However, the study showed some limitations. First, due to the cross-sectional nature of the study design, it was not possible to ascertain a causal relationship between physical activity levels and sleep quality. Second, participants were recruited using a convenience sampling technique, which may introduce selection bias. However, due to the significant time constraints faced by board medical trainees and the limited availability and willingness to participate, convenience sampling was the most practical and feasible approach for this study. Additionally, logistical challenges in accessing a dispersed and highly demanding population further limited the use of probability-based sampling methods. Third, residual confounding cannot be excluded; important variables such as shift duration, number of on-call duties, caffeine intake, and undiagnosed sleep disorders (e.g., obstructive sleep apnea) were not measured. Fourth, the study employed the PHQ-4 scale, which is a valid and reliable screening tool for anxiety and depression, but is not a confirmatory diagnostic tool. Finally, physical activity and psychological symptoms were self-reported, which may introduce reporting bias.

## Conclusions

In this cross-sectional study, anxiety and depression were independently associated with poor sleep quality among Saudi medical residents, while the use of sedative medications was also significantly associated. Residency level and physical activity were not significantly linked to sleep quality after adjustment for potential confounders. These findings highlight that psychological factors may play a more prominent role in sleep disturbances than lifestyle or training-stage variables. Given the study design, causal relationships cannot be inferred. Further longitudinal and interventional studies are needed to clarify the directionality of these associations and to guide strategies for improving sleep quality and overall well-being among Saudi residents.

## Appendices

Questionnaire

Part 1: Demographic and personal characteristics

1. Age: What is your age? (please specify)

2. Height: What is your height? (please specify)

3. Weight: What is your weight? (please specify)

4. Gender: What is your gender?

o    Male

o    Female

5. Marital Status: What is your marital status?

o    Married

o    Separated/Divorced/Widow/Widower

o    Single

6. Pregnancy: Are you currently pregnant?

o    Yes

o    No

o    Not applicable

7. Nationality: What is your nationality?

o    Saudi

o    Non-Saudi

8. Training Sector: What is your training sector?

o    First (Central Region)

o    Second (Western Region)

o    Third (Eastern Region)

o    Fourth (Northern, Al-Madinah, and Al-Qassim Regions)

o    Fifth (Southern Region)

9. Specialization: What is your specialization?

o    Medical Fields

o    Surgical Fields

10. Level of Residency: What is your level of residency?

o    Junior

o    Senior

11. Smoking Status: Please indicate your tobacco smoking status.

o    Current: daily 

o    Current: less than daily

o    Past: daily 

o    Past: less than daily

o    Not at all

12. Caffeine-containing items Consumption Frequency: Over the past month, please indicate how often you consume each of the following caffeine-containing items: Coffee, Tea, Energy drinks, Soda (cola, etc.), Chocolate.

o    1 time in the past month

o    2-3 times in the past month

o    1-2 times per week

o    3-4 times per week

o    5-6 times per week

o    1 time per day

o    2-3 times per day

o    4-5 times per day

o    6 or more times per day

13. Use of Sedative Medications: Do you commonly use any sedative medications?

o    None

o    Melatonin

o    Panadol Night

o    Antihistamines

o    Other (please specify)

14. Medical History: Do you have any chronic diseases or surgical history?

o    Yes, but it does not affect daily activities

o    Yes, and it affects daily activities

o    No

Psychiatric History:

15. Depression Status (PHQ-2): Have you experienced any of the following symptoms over the past 2 weeks?

(Little interest or pleasure in doing things)

o    Not at all

o    Several days

o    More than half the days

o    Nearly every day

(Feeling down, depressed, or hopeless)

o    Not at all

o    Several days

o    More than half the days

o    Nearly every day

16. Anxiety Status (GAD-2): Have you experienced any of the following symptoms over the past 2 weeks?

(Feeling nervous, anxious, or on edge)

o    Not at all

o    Several days

o    More than half the days

o    Nearly every day

(Not being able to stop or control worrying)

o    Not at all

o    Several days

o    More than half the days

o    Nearly every day

17. Treatment Status for Depression and Anxiety: Are you currently receiving treatment for depression or anxiety?

o    Yes

o    No

Part 2: Sleep quality

During the past month:

1. When have you usually gone to bed at night?  Bedtime: (please specify)

2. How long (in minutes) has it usually taken you to fall asleep each night? Number of minutes: (please specify)

3. When have you usually gotten up in the morning? Getting up time: (please specify)

4. How many hours of actual sleep did you get at night? (This may be different than the number of hours you spend in bed.) Hours of sleep per night: (please specify)

5. How often have you had trouble sleeping because you:

(0 = Not during the past month, 1 = Less than once a week, 2 = Once or twice a week, 3 = Three or more times a week).

a. Cannot get to sleep within 30 minutes

Response options:  0 to 3

b. Wake up in the middle of the night or early morning

 Response options:  0 to 3

c. Have to get up to use the bathroom

Response options:  0 to 3

d. Cannot breathe comfortably

Response options:  0 to 3

e. Cough or snore loudly

Response options:  0 to 3

f. Feel too cold

Response options:  0 to 3

g. Feel too hot

Response options:  0 to 3

h. Have bad dreams

Response options:  0 to 3

i. Have pain

Response options:  0 to 3

j. Other (please specify)

Response options:  0 to 3

6. How often have you taken medicine (prescribed or "over the counter") to help you sleep?

o    0: Not during the past month

o    1: Less than once a week

o    2: Once or twice a week

o    3: Three or more times a week

7. How often have you had trouble staying awake while driving, eating meals, or engaging in social activity?

o    0: Not during the past month

o    1: Less than once a week

o    2: Once or twice a week

o    3: Three or more times a week

8. How much of a problem has it been for you to keep up enough enthusiasm to get things done?

o    0: No problem at all

o    1: Only a very slight problem

o    2: Somewhat of a problem

o    3: A very big problem

9. During the past month, how would you rate your sleep quality overall?

o    0: Very good

o    1: Fairly good

o    2: Fairly bad

o    3: Very bad

Part 3: Physical activity

Consider all the activities at work, as part of housework, to get from place to place, and in your spare time for recreation, exercise, or sport.

Vigorous Physical Activities:

I. During the last 7 days, on how many days did you do vigorous physical activities like heavy lifting, digging, aerobics, or fast bicycling?

Response options:  0 to 7 days

II. How much time did you usually spend doing vigorous physical activities on one of those days?

Hours and minutes per day: (please specify)

Moderate Physical Activities:

I. During the last 7 days, on how many days did you do moderate physical activities like carrying light loads, bicycling at a regular pace, or doubles tennis? Do not include walking.

Response options:  0 to 7 days

II. How much time did you usually spend doing moderate physical activities on one of those days?

Hours and minutes per day: (please specify)

Walking:

I. During the last 7 days, on how many days did you walk for at least 10 minutes at a time?

(please specify)

 II. How much time did you usually spend walking on one of those days?

Hours and minutes per day: (please specify)

Sitting:

I. During the last 7 days, how much time did you spend sitting on a weekday?

Hours and minutes per day: (please specify)
